# Managing Vancouver B2 periprosthetic femoral fractures: is open reduction and internal fixation superior to stem revision? A comparative analysis with a minimum 5-year follow-up

**DOI:** 10.1007/s00402-026-06297-1

**Published:** 2026-04-17

**Authors:** Tihui Wang, Hongwei Xu, Jinqing Wu, Xu Wang

**Affiliations:** Mindong Hospital Affiliated to Fujian Medical Univesity, Ningde, China

**Keywords:** Periprosthetic fracture, Vancouver B2 fracture, Open reduction and internal fixation, Stem revision

## Abstract

**Purpose:**

The aim of this study was to compare the long-term outcomes of stem revision (SR) and open reduction and internal fixation (ORIF) for the treatment of Vancouver B2 periprosthetic fractures of the femur.

**Methods:**

From June 2013 to May 2023, 56 consecutive patients were studied at our institution. Four patients were lost to follow-up, four had incomplete data. Thus, 48 cases were included in the analysis. The patients were divided into a stem revision group (SR group with 25 patients) and an open reduction and internal fixation group (ORIF group with 23 patients). The surgical complications, perioperative parameters, and 1-year mortality rates were assessed, the functional outcomes were assessed with the Harris Hip Score (HHS), and the radiographic outcomes were assessed in accordance with the Beals and Tower criteria.

**Results:**

In SR group, the mean follow-up time was 61.2 months, 36% of patients experienced complications, the mean HHS was 75.27, and 92% of the patients had “excellent–good” radiographic outcomes. In ORIF group, the mean follow-up time was 63.7 months, 21.7% of patients experienced complications, the mean HHS was 73.56, and 91.3% of the patients had “excellent–good” radiographic outcomes. The total number of postoperative complications, dislocation rate, blood loss volume, operation time and transfusion rate were lower in ORIF group, and two patients in SR group experienced hip dislocation. There were no significant differences in the 1-year mortality rate, bone healing time and reoperation rate between the two groups. However, while the fracture pattern was considerd, the one zone fracture demonstrated lower radiographic subsidence comparing with two zones fracture (1.18 ± 1.36 mm vs 1.87 ± 1.61 mm). Most of these patients in both groups did not return to their preoperative mobility status.

**Conclusions:**

Although SR is the golden standard for Vancouver B2 periprosthetic fractures of the femur, ORIF can be a viable alternative for frail and low-demand patients on account of signifcantly less perioperative blood loss, shorter operating time and lower medical or total complication rates; expecially for the isolated medial or lateral zone fracture with primary long stem and “happy hips”.

## Introduction

The risk of periprosthetic femoral fracture (PFFs) within 20 years after primary total hip arthroplasty (THA) has been reported to be 3.5% [1], and the incidence of periprosthetic fracture after THA revision is as high as 4–12% [2]. The femoral periprosthetic fractures can occur during surgery or after surgery. PFFs are associated with serious complications which pose challenges for surgeons, including long operating duration, increasing bloodloss, high reoperation rates and mortality. The Vancouver classification [3] is the most common and reproducible classification used for predicting the best treatment for PFFs. The classification is based on fracture type, implant stability and bone quality. Vancouver type B2 PFFs are characterized by a loose stem with good bone stock. Radiographic loosening is subjective, femoral implant stability should be accurately checked in the preoperative planning but is a common experience that the final decision on stem stability is mainly an intraoperative judgement. Some reports underline that up to 20% of loose stems remain undiagnosed at radiographic-based approach and up to 47% of B2 fractures, confirmed intraoperatively, were initially classified as B1 on X-ray evaluation [4, 5].

In previous studies, stem revision (SR) was considered the gold standard for the surgical treatment of Vancouver type B2 fractures [6]. Compared with SR, the open reduction and internal fixation (ORIF) results in a significantly longer bone healing time and less mobility. However, other studies revealed that compared with those of ORIF only, the mid- to long-term results of SR for B2 and B3 PFFs in the elderly patients were satisfactory. Thus, SR is a less invasive procedure with a lower risk of perioperative complications [7]. In recent years, there have been different opinions to treat with the Vancouver B2 PFFs, Which suggested that ORIF may be considered as a viable alternative options in selected cases of Vancouver B2 PFFs [8, 9]. The optimal treatment for type B2 fractures with a loose stem is still unclear. Thus, the aim of this investigation was to compare the functional and radiographic outcomes as well as the complications of SR with those of ORIF for Vancouver B2 fractures.

## Materials and methods

The clinical data of patients who developed Vancouver type B2 PFFs after undergoing THA at our hospital was retrospectively reviewed from the hospital electronic patient system. And the institutional review board by the Ethics Committee of our hospital approved the study. From June 2013 to May 2023, 56 consecutive patients were studied at our institution, the cut-off age was 65 years old. Four patients were lost to follow-up, four had incomplete data. Thus, 48 cases were included in the analysis. These patients were divided into SR group (25 patients) and ORIF group (23 patients). The primary type of fixation for all the patients was cementless. We included only patients with type B2 periprosthetic femur fractures, one zone fracture27 cases (medial zone fracture of 13cases and lateral zone fracture of 14 cases), and two zones fracture (medial and lateral zone fracture) 21 cases. All the patients had complete medical records and radiographic data. Patients with pathological fractures met the exclusion criteria and were followed up for at least 24 months.

The preoperative indication of ORIF for VB2 PFFs included the several points. If the fracture location was at least 1–2 cm proximally to the tip of the femoral prosthesis and do not exceed the apex, the primary femoral prosthesis type was a long stem, patients with ‘happy hips’ before the fracture. The ORIF could be performed. Otherwise, SR was suggested. However, the final decision on stem stability is mainly an intraoperative judgement.

The American Society of Anaesthesiologist (ASA) comorbidity score was recorded to assess comorbidity [10]. The primary diagnosis and fixation type were also assessed. The posterior lateral approach was used for all patients. The length of hospital stay, operation duration and blood transfusion volume were recorded. Radiographs was evaluated by a surgeon who was blinded to the clinical outcome. Bone healing was defined based on callus formation, and the status of bone healing was assessed radiologically on both anteroposterior and lateral radiographs. According to the Beals and Tower criteria for radiological classification [11], outcomes was graded as excellent (a stable implant with minimal deformity), good (a stable implant, minimal or no subsidence, and a well-healed fracture with moderate deformity) or poor (loosening, nonunion, sepsis, severe deformity or new fracture). Implants was considered stable if there were no radiolucent lines around the stem, progressive implant migration, or subsidence [12].

The clinical outcome was assessed based on patient’s mobility classification [13]. Mobility in the period prior to fracture and after fracture healing was categorized as follows (ranging from best to worst): able to walk without help, able to walk with a walking stick, able to walk with a walking frame or two crutches, or unable to walk. While caculating the difference between pre- and postoperative ambulation status, we have set four different level as follows: 1 level increase, no change, 1 level decrease and 2 level decrease. From the electronic records and follow-up office, the follow-up data regarding Harris Hip Score (HHS), radiopraphic datas, perioperative data, complications and reoperations were retrieved.

Re-operation rate was primarily assessed through electronic health records and operative registry in our institutional. Excluding surgeries performed at other hospitals, which was through telephone follow-up or review of outpatient medical records.

### Surgical procedure

**Fig. 1:**
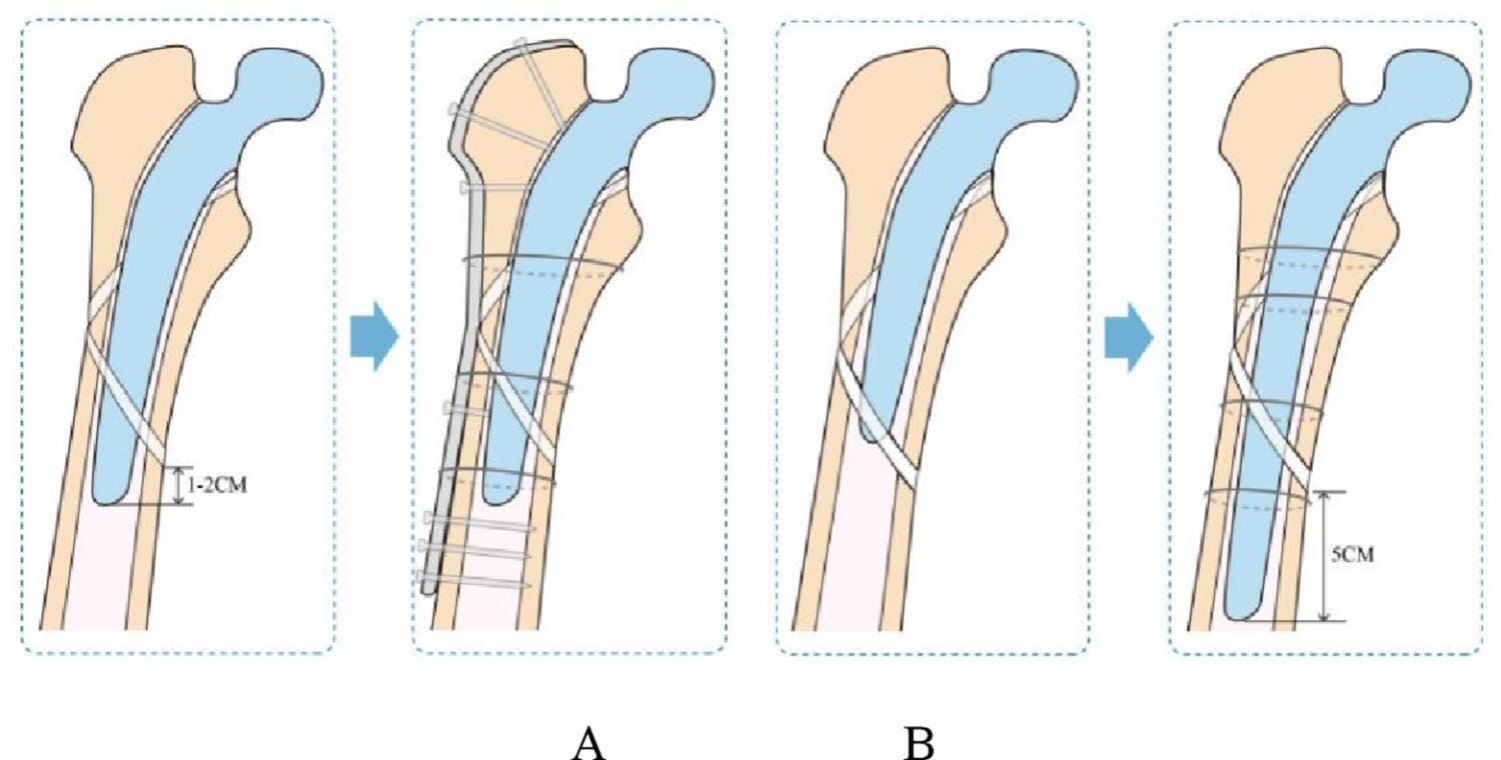
Figure explaining the exact meansurement of the fractured area and the femur prosthesis

All the cases were performed by a same senior arthroplasty surgeon. The posterolateral approach was used for all patients. After exposure the hip, a single hook was used to dislocate the hip joint. Femoral implant stability should be accurately checked in the preoperative planning but was an intraoperative judgement and could only be identified with hip dislocation. The wear of the linner, femoral head and acetabulum stability were assessed. At the meanwhile, the type of fracture and the stability of the femoral prosthesis were evaluated too. If the fracture location was at least 1–2 cm proximally to the tip of the femoral prosthesis and do not exceed the apex, where fracture morphology allowed a secure fixation of the stem. Expecially for the isolated medial or lateral zone fracture with primary long stem and “happy hips”, the ORIF could be performed (Fig. 1A). Otherwise, SR was suggested (Fig. 1B).

In the ORIF group, the fracture segments were reduced by rotating the lower limbs and using two reduction forceps, therefore, 2–4 cerclage wires were frequently used to tie up the fracture. Then, the claw-shaped steel plate (Dabo, Beijing, China) hooked the tip of the greater trochanter of the femur, which bypassed the segments of the femur with at least eight cortices. After plate fixation, the reliability of fracture fixation and joint stability were evaluated. Then reducing the hip joint, reconstructing the external rotator muscle group, and closing the incision.

In SR group, Due to the personal habits of the surgeons, the tapered fluted modular titanium stem (SL; AiKang, Beijing, China) was choosen for all the patients. Firstly, the stem was carefully removed without causing additional iatrogenic fracture, before expanding the medullary cavity, 1 to 2 steel wires were pre-bound to the distal part of the fracture. Then, gradually expand the femoral marrow cavity from small to large using femoral reamers, it was considered the femoral prosthesis size was suitable until the internal and external rotation of the femoral test mold could drive the distal part of femur. Then the proper size of tapered fluted modular titanium stem was implanted at least 5 cm below the fracture [14]. And then, the appropriate neck length and femoral head diameter were choosen to ensure the stability of the hip joint. After that, the fracture was subsequently reduced anatomically and fixed using wires or cables. Then reconstructing the external rotator muscle group, and closing the incision.

### Post-operative treatment

The rehabilitation program was carried on case by case, considering bone quality, shape of fracture, age and comorbidity of single patient. In general, a partial bearing (50%) with crutches or walker was adopted from the first postoperative day after ORIF until the bone healed. In the SR group, a full weight-bearingwas performed from the first postoperative day. Antibiotic prophylaxis with Cephalosporin and standard venous thromboembolism prophylaxis were administered routinely. In all case 1 drain was used and removed within the second post-operative day. In the outpatient clinic, patients were scheduled for routine follow-up, the HHS scores was used to evaluate functional outcomes in 1, 3, 6, 9 and 12 months postoperatively, annualy until the last follow-up.

### Statistical analysis

For continuous quantitative data, if they conform to a normal distribution, the Mean ± SD deviation is used for description, and the T-test is used for comparison between two groups; if they do not conform to a normal distribution, the median [P25, P75] is used for statistical description, and the rank sum test is used for comparison between groups. For count data, the number of cases (%) is used for description, and the Chi-Square test or Fisher's exact probability method is used for comparison between groups. Considering that this study is not an RCT study and there are the influences of confounding factors, we further used the MatchIt package in R language to perform 1:1 propensity score matching (nearest neighbor matching, with a caliper value of 0.2). The confounding variables included in the matching were Age and BMI. Additionally, we used the WeightIt package in R language to conduct inverse probability weighting to further analyze the confounding factors, with the weighting variables being the same as those in the aforementioned PSM. The balance between groups before and after matching or weighting was evaluated using the standardized mean difference (SMD value). When the SMD value decreased after PSM and was less than 0.2, it was considered that the balance between groups was good. For the data after PSM or weighting, the above analyses were repeated, and the weights were considered in the weighted analysis. All statistical analyses and related chart drawing were conducted in R language (version 4.4.1), and a two-sided P < 0.05 was considered statistically significant.

## Results

### Demographic characteristics

The distribution of patients’characteristics was showed in Table 1. There was no statistically significant correlations with the ASA score, implant type, reason for primary arthroplasty, fracture pattern, time-to-fracture, sex, age and BMI between the two surgical groups (p > 0.05) (Table 1).

**Table 1:** Patient characteristics

Parameters	Before matching, Mean (x ± s), n (%)			After matching, Median (Q1, Q3); n (%)		
	SR (n = 25)	ORIF (n = 23)	P value	SR (n = 24)	ORIF (n = 22)	P value
Follow-up (m)	61.2 ± 12.6	63.7 ± 14.3	0.324	61.00 (57.00,67.00)	64.00 (60.00,69.00)	0.131
Age (years)	72.8 ± 11.4	74.3 ± 10.8	0.152	72.00 (70.00,76.00)	75.00 (70.00,79.00)	0.563
BMI (kg/m^2^)	24.6 ± 2.5	23.5 ± 3.1	0.221	24.10 (21.00,26.00)	24.00 (20.00,25.80)	0.482
Sex (n)			0.451			0.636
Male	10 (40)	9 (39.2)		15 (65)	12 (58)	
Female	15 (60)	14 (60.9)		8 (35)	9 (42)	
ASA score (n)			0.231			0.149
1	1 (4)	0 (0)		1 (2.2)	0 (0)	
2	16 (64)	14 (60.8)		14 (59)	13 (60)	
3	7 (28)	6 (26.1)		9 (37)	4 (21)	
4	1 (4)	3 (13.0)		1 (2.4)	4 (19)	
Reason for primary arthroplasty (n)			0.405			0.569
Fractures of the femoral neck only	12 (48)	13 (56.5)		13 (56)	10 (46)	
Osteoarthritis	13 (52)	10 (43.5)		10 (44)	12 (54)	
Implant type of primary THA			0.350			0.293
Hemiarthroplasty	12 (48)	11 (47.8)		13 (56)	8 (39)	
Total hip arthroplasty	13 (52)	12 (52.2)		10 (44)	13 (61)	
Fracture pattern			0.971			0.619
One zone fracture	14 (56)	13 (56.5)		12 (51)	13 (59)	
Two zones fracture	11 (44)	10 (43.5)		11 (49)	9 (41)	
Time-to-fracture (m)	41.5 ± 10.2	38.6 ± 11.5	0.180	39.00 (35.00,46.00)	40.00 (36.00,48.00)	0.874

The values was presented as the mean value and standard deviation. ORIF, Open Reduction and Internal Fixation; SR, Stem Revision; BMI, Body Mass Index; ASA, American Society of Anaesthesiologists. Mean (x ± s), Median (Q1, Q3); n (%),* Represent P < 0.05

### Perioperative parameters

The ORIF group comparing with the SR group demonstrated superior outcomes in multiple perioperative parameters, including less intraoperative blood loss (612.2 ± 173.0 mL vs 1100.8 ± 200.4 mL), shorter operative time (104.5 ± 30.7 min vs 136.2 ± 48.5 min), lower volume of RBCs transfused (106.1 ± 24.5 mL vs 251.5 ± 30.2 mL) and shorter hospital stay time (10.5 ± 3.0 days vs 14.1 ± 5.7 days) (Table 2).

**Table 2:** Perioperative parameters and clinical outcomes

Parameters	Before matching, Mean (x ± s), n (%)			After matching, Median (Q1, Q3), n (%)		
	SR (n = 25)	ORIF (n = 23)	P value	SR (n = 24)	ORIF (n = 22)	P value
Operation duration (min)	136.2 ± 48.5	104.5 ± 30.7	0.012*	130.00 (95.00, 168.00)	100.00 (90.00, 115.00)	0.004*
Intraoperative blood loss volume (ml)	1100.8 ± 200.4	612.2 ± 173.0	0.022*	1,150.00 (950.00, 1,400.00)	650.00 (550.00, 1,000.00)	< 0.001*
Volume of RBCs transfused (ml)	251.5 ± 30.2	106.1 ± 24.5	0.025*	230.00 (0.00, 400.00)	110.00 (0.00, 200.00)	0.032*
Hospital stay (days)	14.1 ± 5.7	10.5 ± 3.0	0.031*	14.00 (11.00, 18.00)	12.00 (9.00, 15.00)	0.129
Harris hip score (HHS)	75.27 ± 10.51	73.56 ± 11.27	0.212	74.00 (73.00, 78.00)	75.00 (72.00, 78.00)	0.581
Beals and tower classification			0.524			0.947
Excellent	10 (40)	11 (47.8)		10 (44)	9 (41)	
Good	13 (52)	10 (43.5)		12 (49)	11 (49)	
Poor	2 (8)	2 (8.7)		2 (6.7)	2 (9.4)	
Difference between pre- and postoperative ambulation status			0.461			0.086
No change	0	0		7 (29)	10 (48)	
1 level increase	9 (36)	8 (34.8)		12 (49)	11 (49)	
2 level decrease	12 (48)	14 (60.9)		5 (22)	1 (2.3)	
Dislocation (%)	2 (8)	0 (0)	0.005	2 (6.6)	0 (0)	0.213
Dislocation without surgery (%)	1 (4)	0 (0)	0.012	1 (3.3)	0 (0)	
Deep infection (n) (%)	1 (4)	0 (0)	0.012	1 (3.3)	0 (0)	0.352
Radiographic subsidence (mm)	1.14 ± 1.07	1.37 ± 1.52	0.186	1.00 (0.80, 1.30)	1.00 (0.85, 1.40)	0.869
Subsidence more than 2 mm (n) (%)	5 (20)	6 (26.1)	0.250	4 (16)	5 (22)	0.646
Reoperation rate (%)	2 (8)	2 (8.7)	0.373	2 (6.7)	1 (6.0)	0.910
Medical complications (n) (%)	6 (24)	2 (8.7)	0.032*	6 (25)	4 (17)	0.036*
Total complications (n) (%)	9 (36)	4 (17.4)	0.028*	7 (32)	5 (23)	0.042*
One-year mortality (%)	3 (12)	3 (13.1)	0.531	3 (12)	2 (8.7)	0.671

Mean (x ± s), Median (Q1, Q3); n (%),* Represent P < 0.05

### Clinical outcomes and complications

**Fig. 2:**
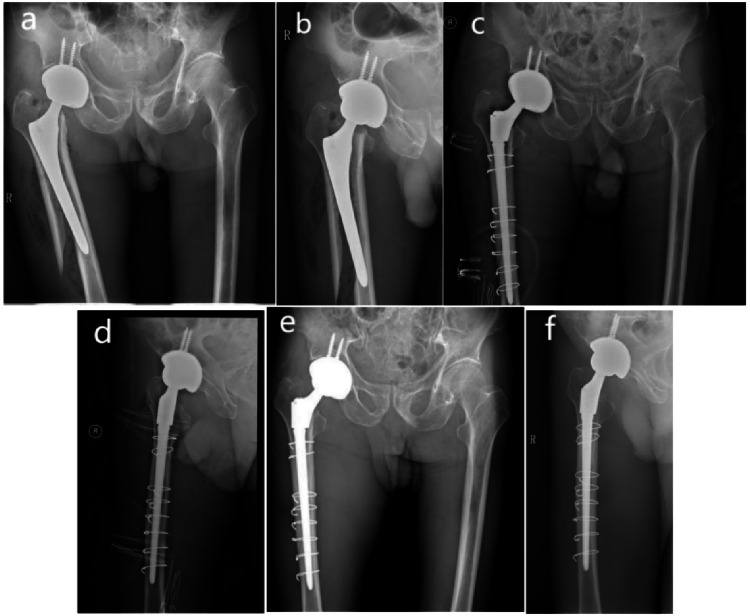
Radiographic examination of a Vancouver B2 PFFs of the right femur in a 78-year-old male (**a**, **b**) treated by simple femur revision with modular femoral stem and wires. The postoperative radiograph showed an anatomic reduction (**c**, **d**). The radiograthic examination taken at follow-up, 6 years later, showed a fracture healing without deformity or shortening (excellent result,according to Beals and Tower’s criteria) (**e**, **f**). At clinical evaluation, the HHS scored 90 points

In the SR group (Fig. 2), the mean follow-up was 61.2 months (24–117 months). the mean HHS was 75.27, and 92% of the patients had “excellent–good” radiographic outcomes. A significant deterioration of ambulation was found after these fractures and 16 patients (64%) had not regained their prefracture walking status at the most recent follow-up. 10 patients who were able to walk without help preoperatively, 8 patients (32%) had decreased 1 level of prefracture walking status to walk with a walking stick, and 2 patient (8%) had decreased 2 level to walk with two crutches postoperatively. 6 patients (24%) who were able to walk with a walking stick, and had decreased 1 level to walk with a walking frame or two crutches, and 2 patient (8%) had decreased 2 level to walk with two crutches postoperatively. Thirty-six percent of patients in the SR group experienced complications, and the reoperation rate was 8% (Table 2). One patient (4%) developed a deep infection within 3 weeks after surgery. A DAIR (debridement, antibiotics and implant retention) procedure was performed in combination with a 6-week course of antibiotics, which successfully eradicated the infection. Two patients (8%) in the SR group experienced hip dislocation, First one was a 72 years female. experienced recurrent posterior dislocation at 3 months postoperatively, attributed to extensive soft tissue dissection and unclear anatomical signs of proximal femur, during the operation, the polyethylene liner was adjusted, with no further episodes of dislocation. Another was a 65 years male experienced recurrent anterior dislocation at 3 months postoperatively, attributed to the gluteus medius insufficiency and primary antero-lateral surgical approach. Closed reduction was performed and a brace was used for 6 weeks, there was no signs of dislocation recurrence at 5 years of follow-up. Medical complications occurred in 24% of patients (n = 6): two renal failure, and four postoperative pneumonia. The 1-year mortality for SR patients was 12%.

**Fig. 3:**
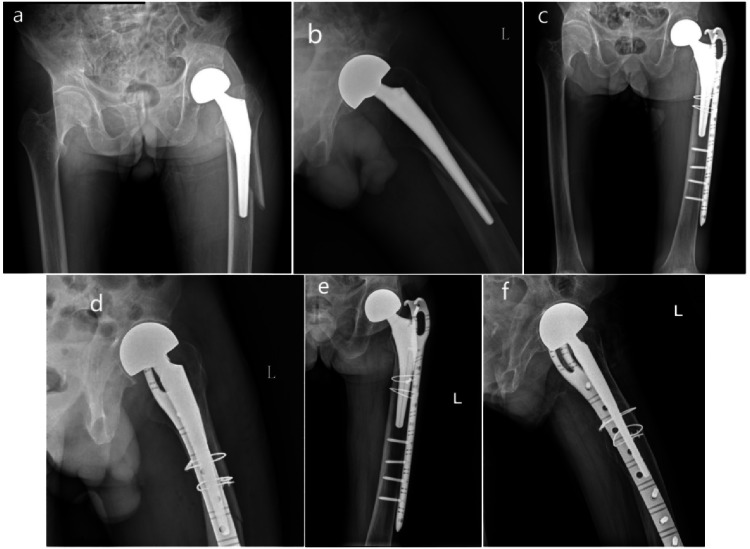
Radiographic examination of a Vancouver B2 PFFs of the left femur in a 83-year-old male (**a**, **b**) treated by ORIF. The postoperative radiograph showed an anatomic reduction (**c**, **d**). The radiograthic examination taken at follow-up, 4 years later, showed normal fracture healing with stem subsidence about 4 mm (good result, according to Beals and Tower’s criteria) (**e**, **f**). At clinical evaluation, the HHS scored 80 points

In the ORIF group (Fig. 3), the mean follow-up time was 63.7 months (24–120 months), the mean HHS was 73.56, and 91.3% of the patients experienced “excellent-good” radiographic outcomes. A significant deterioration of ambulation was found after fractures, and 15 patients (65.2%) had not regained their prefracture walking status at the most recent follow-up. 9 patients who were able to walk without help preoperatively, 8 patients (34.8%) had decreased 1 level of prefracture walking status to walk with a walking stick, and 1 patient (4.3%) had decreased 2 level to walk with two crutches postoperatively. 5 patients (21.7%) who were able to walk with a walking stick, and had decreased 1 level to walk with a walking frame or two crutches postoperatively. In this study, there were 13 cases of one zone fracture and 10 cases of two zones fracture in the ORIF group; while 14 cases of one zone fracture and 13 cases of two zones fracture in the SR group. In the ORIF group. The one zone fracture demonstrated lower radiographic subsidence comparing with two zones fracture (1.18 ± 1.36 mm vs 1.87 ± 1.61 mm). A total of 21.7% of patients experienced complications, and the reoperation rate was 8.7%. Two patients with two zones fracture in the ORIF group received stem revision due to the mechanical failure. The first one was a 72 years female with severe osteoporosis experienced 7 mm radiographic subsidence of femoral prosthesis combined with thigh pain and mechanical failure in the 5 months postoperatively, and received stem revision at 14 months after surgery. The second was a 75 years male with severe osteoporosis and obesity experienced mechanical failure with 9 mm radiographic subsidence in the 6 months postoperatively, received stem revision at 26 months after surgery. The 1-year mortality rate for patients who underwent ORIF was 13.1% (Table 2).

The total postoperative complication rate and dislocation rate were lower in the ORIF group. There were no significant differences in the 1-year mortality rate, bone healing time and reoperation rate between the two groups. Whatever treatment was adopted, there was an overall worsening of quality of life. This worsening of postoperative ambulatory status was not statistically significant (p = 0.461). The mean HHS at the most recent follow-up was not statistically significant (p = 0.212). According to the Beals and Tower classification, the two surgeries had relatively similar results (p = 0.765) (Table 2).

### Typical cases

See Figs. 2, 3.

## Discussion

The incidence of periprosthetic femoral fractures has increased with the aged patients, while the number of total hip arthroplasty has been increasing year by year [15, 16]. Periprosthetic femoral fractures (PFFs) can occur intraoperatively or postoperatively. PFFs are associated with serious complications, such as long operating times, an increased risk of bleeding, high reoperation rates and mortality, especially in elderly patients, thus posing challenges for orthopaedic surgeons [17, 18], because the surgical management requires expertise both in fracture fixation and in revision arthroplasty. Moreover, since these fractures usually occur in elderly population with severe osteoporosis [19], a comprehensive evaluation of the patients is crucial to choose the most tailored treatment. A complete and multidisciplinary evaluation including fracture pattern, previous situation of THA, type of stem, medical comorbidities and bone quality is necessary [20].

Although there is now more evidence to describe the treatment of Vancouver B fractures, there is still limited knowledge regarding factors that cause surgeons to perform either an ORIF or SR. In this study, the femoral implant stability can only be identified with hip dislocation intraoperatively. If the fracture location was at least 1–2 cm proximally to the tip of the femoral prosthesis and do not exceed the apex, where fracture morphology allowed a secure fixation of the stem. expecially for the isolated medial or lateral zone fracture with primary long stem and “happy hips”, the ORIF could be performed. Otherwise, SR was suggested. Spina [21] stated that the straight stem has a higher fixation in the diaphyseal part of the femur, the fixation of the stem into the diaphyseal canal may not be completely lost while the fracture does not exceed the apex. We choosed the ORIF as the fracture location was at least 1–2 cm proximally to the tip of the femoral prosthesis and do not exceed the apex. Baum [22] stated that the Vancouver classification did not distinguish between fracture patterns. In our study, althought there was no significant differences in radiographic subsidence between ORIF and SR, while the fracture pattern was considerd, the one zone fracture demonstrated lower radiographic subsidence comparing with two zones fracture (1.18 ± 1.36 mm vs 1.87 ± 1.61 mm, p < 0.05). Two patients with two zones fracture in the ORIF group received stem revision due to the mechanical failure. An anatomical reduction is mandatory for VB2 PPFs. Fractures with a single fragment could be anatomically reduced and fixed using ORIF. While multiple or comminuted fractures make osteosynthesis complicated, SR would be a more suitable option. As it is sufficient to bring the fragments closer together without the need for anatomical reduction. David [23] proposed a subclassification of VB2 PPFs that the proximal femur was divided into the lateral, medial and distal zone. In this study, fractures were categorized into one zone fracture (medial or lateral zone fracture) and two zones fracture (medial and lateral zone fracture). We did not choose the distal zone fracture as the subgroup, because SR was require if the fracture have exceeded the apex of the femur stem. Eckardt [24] held that the main criteria for using ORIF in our institution is a periprosthetic fracture with a maximum of 3–4 large fragments that could be reconstructed anatomically. David [23] concluded that V-B2 PFFs treated ORIF affecting only one zone (medial, lateral, or distal) have a lower risk of complication than those affecting two or more zones. Therefore, while the fracture pattern was considerd, ORIF was more suitable for isolated medial or lateral fracture without exceeding the apex of the femur prosthesis, expecially for the primary long stem and “happy hips”.

The Swedish National Hip Arthroplasty Register showed a reoperation rate of 32% in patients treated with ORIF alone compared to a 10% and 23% rate in patients managed with stem revision alone or stem revision and ORIF. Stefano [25] showed that six (5.3%) patients developed new traumatic PPFs without evidence of radiologic stress shielding or stem loosening; and 4 displacements of fracture (2 at 30 days and 2 at 90 days after the indexed procedure) that required reoperation. In our study, 21.7% of patients experienced complications in the ORIF group, and the reoperation rate was 8.7%. Two patients with two zones fracture in ORIF group experienced 7 and 9 mm radiographic subsidence of femoral prosthesis combined with thigh pain, received revision because of mechanical failure in the 14 and 26 months after surgery, respectively. The potential risk factors were severe osteoporosis and obesity. However, the retrospective design of this study did not allow for a standardized analysis of preoperative femoral bone quality or the management of osteoporosis. Victoria [19] showed that patients with PFFs had less frequent T-score osteoporosis, advanced age and higher BMI than patients with native hip fractures. Although it would be very interesting to know whether the radiographic subsidence of prosthesis was associated with weight-bearing, there is poor available evidence. Thomas [26] found a significantly higher rate of subsidence in the ORIF compared with SR, however, no significant difference in terms of loosening was observed. H Eckardt [24] observed 97 VB2 PFFs (68 cases of cementless and 29 cases of cement) with a mean follow-up of 347 days in the ORIF group and 586 days in the SR group, and showed that the reason for failure in the ORIF were infection (*n* = 5), persistent symptomatic stem loosening (*n* = 3) and refracture (*n* = 3) after a new fall. Jains [27] showed that the 2-year complication rate was higher after SR (25% vs. 10%) with hip dislocation being the most common complication. Stefano [25] showed five (4.4%) dislocation were observed. In particular, 3 dislocations occurred during the hospitalization of patients, and in two cases an acetabular revision was performed, using a dual mobility cup; 2 episodes happened at more than 45 days from surgery, were referred as movements exceeding the usual range of motion, and were treated conservatively with good final outcome. In our study, two patients (8%) in the SR group experienced hip dislocation (a patient in one zone fracture and another in two zones fracture). It was the most frequent complication of SR, The main risk of dislocation postoperation in this study were extensive soft tissue dissection, unclear anatomical signs of proximal femur, and the gluteus medius insufficiency. Showed no recurrent dislocations after adjusting the polyethylene liner or closed reduction followed by 6 weeks of brace immobilizationand. Accurate preoperative planning for the measurements of the prosthetic components, including the dimensions of the prosthetic stem, is essential during the treatment of Vancouver B2 periprosthetic fractures with SR to reduce the risk of further complications such as dislocations or periprosthetic refractures [28]. In the previous study, the author had measured and marked the anatomical location of the contralateral lower limb to improve the accurate placement of the prosthesis [29]. Additionally, the primary surgical approach and soft tissue protection during the operation may help preventing hip dislocation after surgery.

Some authors have suggested that in patients with Vancouver B2 fractures who undergo SR or ORIF, The demand to maintain a particular level of function and the risk of anaesthesia of the patients should be considered [30]. Since the majority of the patients are old and affected by multiple comorbidities, early mobilization and a rapid return to self-reliance and independence are crucial to prevent further adverse events and avoiding stasis-related complications [31]. Sagi [32] showed that patients chosen for ORIF were significantly older than those treated by SR (85.4 vs 75.1 years), blood loss was 390.7 (ORIF) and 1141.6 ml (SR), surgical times were 134.5 (ORIF) and 225 min (SR). In our study, the medical complication rates were 8.7% for ORIF and 24% for SR, This maybe related to the shorter operation time (104.5 min of ORIF and 136.2 min of SR) and lower blood loss volumes (612.2 ml of ORIF and 1100.8 ml of SR). Fei [33] also found that the average age in the ORIF group was higher than that in the SR group (81 years vs. 78.4 years). They speculated that ORIF is chosen because elderly patients with PFFs often have multiple comorbidities, which typically excluded highly complex surgeries such as SR. Anna [34] showed that patients with an ASA score > 3 underwent ORIF more frequently, and the mean age was 85 years old. In our study, most of those patients are ASA 2, maybe attribute to relatively younger age comparing with the previous studies (74.3 years of ORIF and 72.8 years of SR).

This study has several limitations. First, the relatively small sample size from a single-center may decrease its statistical power to detect significant differences in outcomes and complications. Second, the retrospective design did not allow for a standardized analysis of preoperative femoral bone quality or the management of osteoporosis. And the relationship between the radiographic subsidence and the weight-bearing timing. Third, due to surgeon preference, a tapered fluted modular titanium stem was used exclusively in the SR group. This precluded comparisons between cemented and cementless stems or between modular and non-modular designs; however, it also enhanced the internal comparability between the ORIF and SR groups. Finally, radiologic landmarks were some what ambiguous and depend on the patient’s exact positioning, such as hip flexion or extension and internal or external rotation. Further high-quality long-term studies are expected to explore these unsolved problems.

## Conclusions

We reported comparable radiographic and clinical results of ORIF and SR for the treatment of Vancouver B2 periprosthetic fractures. Althought good to excellent radiographic outcomes and pain relief is achievable, only 34.8% (8/23) in the ORIF group and 36% (9/25) in the SR group recoverd to their pre-fracture walking status. Admiting that SR is the golden standard for Vancouver B2 periprosthetic fractures of the femur, ORIF can be a viable alternative for frail and low-demand patients on account of signifcantly less perioperative blood loss, shorter operating time and lower medical or total complication rates; expecially for the isolated medial or lateral zone fracture with primary long stem and “happy hips”.

## Data Availability

The datasets generated during and/or analyzed during the current study are not publicly available, but are available from the corresponding author on reasonable request.
